# A Rapid Educational Intervention to Enhance Brain MRI Interpretation Skills of Radiology Professionals for Dementia Diagnosis in Uganda: A Pre‐ and Post‐intervention Study

**DOI:** 10.1002/alz70856_098173

**Published:** 2025-12-24

**Authors:** Rita Nassanga, Noeline Nakasujja, Martha Sajatovic, Michael Grace Kawooya, Steve Jones, Mark Kaddumukasa

**Affiliations:** ^1^ Makerere University, College of Health Sciences, Kampala, Kampala, Uganda; ^2^ Makerere University, Kampala, Central, Uganda; ^3^ University Hospitals Cleveland Medical Center, Cleveland, OH, USA; ^4^ Ernest Cook Ultrasound and Research Education Centre, Kampala, Kampala, Uganda; ^5^ Imaging Institute, Cleveland Clinic, Cleveland, OH, USA

## Abstract

**Background:**

Accurate interpretation of brain magnetic resonance imaging (MRI) data is vital for diagnosing dementia, as it can guide physicians toward proper diagnosis and management. In low‐income settings such as Uganda, the challenges are greater due to limited diagnostic resources and gaps in radiologists' skills. Brain MRI is rarely used in dementia diagnosis, and even when it is employed, subtle but critical findings, such as mild regional atrophy, are often overlooked. This highlights the pressing need to enhance the MRI interpretation skills of imaging practitioners, ensuring that essential diagnostic clues are not missed in patients with suspected dementia.

The main objective was to determine the effect of a training intervention on the brain MRI interpretation skills of radiologists and radiology residents among patients with a clinical diagnosis of dementia.

**Methods:**

This study used a pre‐ and post‐test design involving practicing radiologists and radiology residents. The participants first completed a baseline assessment to evaluate their knowledge and skills before attending a one‐day intensive training workshop led by expert neurologists and radiologists. One month later, they underwent a post training assessment identical to the baseline assessment. Data from both assessments were analysed via STATA version 17.0, and a one‐sided Mann‒Whitney U test was employed to evaluate changes in theoretical knowledge and radiology image reporting skills following the educational intervention.

**Results:**

Thirty one participants completed the baseline assessment, training assessment and post training assessment. Of these, 18 were female. There were 17 radiologists and 14 radiology residents with varying years of experience in brain MRI interpretation. There was a significant increase in both theoretical knowledge (*p* <0.001) and skills in image interpretation (*p* <0.001) after training. Compared with those with more years of experience, those with fewer years of experience in MRI demonstrated greater increases in knowledge and skills.

**Conclusion:**

The participants demonstrated significant improvement in both knowledge and skills in brain MRI interpretation in the diagnosis of dementia following training. The study thus underscores the critical importance of targeted educational programs in enhancing the ability of imaging practitioners to accurately interpret brain MR images for patients with clinically suspected dementia.

